# Coexistence of iAMP21 and *ETV6-RUNX1* fusion in an adolescent with B cell acute lymphoblastic leukemia: literature review of six additional cases

**DOI:** 10.1186/s13039-016-0294-0

**Published:** 2016-11-21

**Authors:** Jun Gu, Alexandra Reynolds, Lianghua Fang, Corrie DeGraffenreid, Kenneth Sterns, Keyur P. Patel, L. Jeffrey Medeiros, Pei Lin, Xinyan Lu

**Affiliations:** 1 School of Health Professions, The University of Texas MD Anderson Cancer Center, 1515 Holcombe Blvd. Unit 0002, Houston, TX 77030 USA; 2 Department of Hematopathology, The University of Texas MD Anderson Cancer Center, 1515 Holcombe Blvd. Unit 0350, Houston, TX 77030 USA; 3 Department of Hematopathology, The University of Texas MD Anderson Cancer Center, 1515 Holcombe Blvd. Unit 0149, Houston, TX 77030 USA; 4 Department of Oncology, Jiangsu Hospital of Traditional Chinese Medicine, Nanjing, Jiangsu China; 5 Department of Hematopathology, The University of Texas MD Anderson Cancer Center, 1515 Holcombe Blvd. Unit 0072, Houston, TX 77030 USA; 6 Department of Pathology, Northwestern University Feinberg School of Medicine, 303 East Chicago Avenue, Tarry 7-723, Chicago, IL 60611 USA

**Keywords:** B-ALL, iAMP21, *RUNX1* amplification, *ETV6-RUNX1* fusion, SNP microarray

## Abstract

**Background:**

Intrachromosomal amplification of chromosome 21 (iAMP21) results from breakage-fusion-bridge cycles and chromothripsis is a distinct marker of a subgroup of B cell acute lymphoblastic leukemia (B-ALL) cases associated with a poor prognosis. iAMP21 accounts for 2% of pediatric B-ALL and occurs predominantly in older children or adolescents. *ETV6-RUNX1* fusion, resulting from t(12;21)(p13;q22), is associated with an excellent outcome in younger children with B-ALL. Coexistence of iAMP21 with *ETV6-RUNX1* fusion is extremely rare with limited clinical information available.

**Results:**

We report the case of an 18-year old Caucasian man diagnosed with *ETV6-RUNX1* fusion positive B-ALL. He was treated with intensive chemotherapy and achieved remission for 6 months before relapse, 15 months after the initial diagnosis. G-band karyotyping and Fluorescence in situ hybridization (FISH) analyses performed on bone marrow revealed complex abnormalities: 41,X,-Y,der(3)t(3;20)(p11.2;q11.2),-4,t(5;22)(q32;q11.2),del(9)(p13),dic(9;17)(p13;p11.2),t(12;21)(p13;q22),der(14)t(14;17)(p11.2;q11.2),der(17;22)(q11.2;q11.2),-20,add(21)(q22),-22[4]/46,XY[15] with an iAMP21 and an *ETV6-RUNX1*. Additional molecular studies confirmed *ETV6-RUNX1* fusion and with a *TP53* mutation. High-resolution single nucleotide polymorphism microarray (SNP array) revealed the iAMP21 to be chromothripsis of 21q and subsequent metaphase FISH further delineated complex genomic aberrations. Although the patient received intensive chemotherapy with allogenic stem cell transplant, he died 26 months after initial diagnosis. We searched the literature and identified six cases showing coexisting iAMP21 and *ETV6-RUNX1*. The median age for these six patients was 10 years (range, 2–18) and males predominated. The median overall survival (OS) was 28 months.

**Conclusions:**

Patients with B-ALL associated with both iAMP21 and *ETV6-RUNX1* tend to be older children or adolescents and have a poor prognosis.

## Background

The latest revision to the World Health Organization (WHO) classification of B-cell lymphoblastic leukemia/lymphoma (B-ALL) has added B-ALL with intrachromosomal amplification of chromosome 21 (iAMP21) as an entity in the group of B-ALL with recurrent genetic abnormalities [1]. iAMP21 is a distinct marker that can be readily detected by metaphase FISH [2] and is caused by breakage-fusion-bridge cycles and chromothripsis, which is a phenomenon reported in cancer genomes, resulted from tens to hundreds of genomic rearrangements occur in a cellular crisis. Chromthripsis can involve one or more chromosomes, often with massive copy number aberrations [3]. Recent study suggested that hyperploidy and telomere attrition could be triggering events for chromothripsis and are frequently associated with *TP53* mutation [4].

B-ALL associated with iAMP21 is a poor prognostic subgroup that represents 2% of pediatric B-ALL cases. The median age of patients is 9 years old and there is a prevalence of males. Patients with iAMP21 often show low platelet and low white blood cell counts (WBC) [5–8]. These patients have a relapse rate that is three times higher than other B-ALL patients are and therefore patients often require intensified therapy, particularly in older children or adolescents with B-ALL [9].

The t(12;21)(p13;q22) which results in the formation of the *ETV6-RUNX1* fusion gene accounts for about 25% of pediatric B-ALL. Patients with B-ALL associated with *ETV6-RUNX1* tend to be younger children and patients have a favorable outcome [10]. iAMP21 has been reported rarely in B-ALL associated with *ETV6-RUNX1* [11].

In this study, we describe a patient with B-ALL associated with both iAMP21 with *ETV6-RUNX1* that we have characterized extensively by using molecular and cytogenetic methods. We also reviewed the literature and identified six similar cases [7, 12]. This combination of molecular alterations in B-ALL tends to occur in older male patients who have a poor prognosis.

## Results

The patient was an 18-year old Caucasian man who presented initially with pancytopenia. A complete blood count showed: WBC 2.0 × 10^9^/L, platelets 88 ×10^9^/L and hemoglobin 8.3 g/dL. Bone marrow examination showed 61% blasts and the patient was diagnosed with a B-ALL at another institution (Table 1). FISH studies performed on the bone marrow aspirate smears showed *ETV6-RUNX1* fusion in 28% of interphases with no evidence of *BCR-ABL1* or *MLL* gene rearrangements. No concurrent chromosome data were available from the initial bone marrow studies. The patient did not have central nervous system (CNS) involvement and he was treated with the intrathecal cytarabine, daunorubicin, vincristine, intrathecal methotrexate, PEG asparaginase and prednisone (CALGB 10403 regimen) elsewhere. The patient did not respond well initially although he eventually achieved remission for 6 months after a second round of chemotherapy. The patient then began to show minimal residual disease by flow cytometry immunophenotypic analysis 8 months after the initial diagnosis, and eventually relapsed 15 months after the diagnosis. The patient was transferred to our institution at this time (Table 1).

**Table 1:** Clinical and laboratory data of the patient

Date	10/9/2013	6/24/2014	12/9/2014	12/31/2014	6/8/2015	8/19/2015
Significant event	Initial diagnosis	MRD Positive	1^st^ Relapse	Persistent disease	Post-ASCT^a^	2^nd^ Relapse
BM Blast (%) (0–5)	61	NA	5	54	1	25
WBC (x10^9^/L)	2.0	5.5	4.6	2.8	5.6	2.2
PLT (x10^3^/μL)	88	383	202	79	133	79
HB (g/dL)	8.3	10.8	13.3	12.9	9.1	11.4
Flow-cytometry analysis	Positive^b^	Positive	Positive^c^	Positive^d^	NA	Positive
G-Band karyotyping	NA^e^	NA	46,XY	Complex	46,XY	46,XY
*ETV6-RUNX1/iAMP21* FISH	Positive	Negative	Borderline Negative	Positive	NA	NA
*ETV6-RUNX1* by PCR	NA	NA	NA	Positive	NA	NA
Treatment	CALGB^f^	Post- CALGB	Blinatumomab	Hyper-CVAD^g^ + Inotuzumab	50 days Post-ASCT	EPOCH^h^ + Rituxan

^a^ *Post ASCT* post allogeneic stem cell transplantation

^b^CD19lo, CD22+, cytoplasmic CD79a+, HLA-DR+, aberrant CD13 and CD33 expression, CD3-, CD10-, CD20-, surface Ig-, CD34-, CD38-,MPO-

^c^CD10+, CD13+, CD19lo, CD33+/lo, CD9-, CD20-, CD34-, surface Ig-

^d^CD10+, CD13+, CD19+, CD22+,CD33+ (subset), CD45 dim, CD38 dim, CD58, cytoplasmic CD79a, CD81, TdT+, cytoplasmic CD3-, CD15-, CD20-, CD25-, CD34-, CD66c-, CD117-, CRLF2-, cytoplasmic IgM-, MPO-

^e^ *NA* not available

^f^CALGB: IT Cytarabine, Daunorubicin, Vincristine, IT Methotrexate, PEG Asparaginase and Prednisone

^g^Hyper-CVAD: Cyclophosphamide, Vincristine, Doxorubicin Hydrochloride, Dexamethasone

^h^EPOCH: Etoposide, Vincristine, Cyclophosphamide, Doxorubicin Hydrochloride

**Fig. 1:**
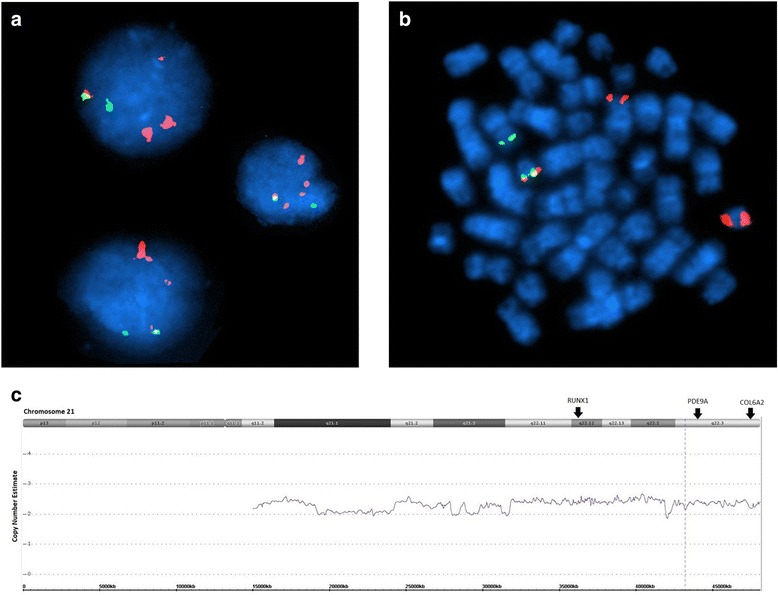
Interphase FISH, metaphase FISH, and SNP microarray analyses. **a** Interphase FISH showed iAMP21 and *ETV6-RUNX1* fusion. **b** Metaphase FISH indicated a derivative chromosome 21 with *ETV6-RUNX1* fusion, an iAMP21, and a derivative 12 with a single *RUNX1* signal. **c** SNP microarray showing the chromothripsis-like pattern of chromosome 21q11.2-21q22.3 (15,006,457 – 48,097,372)

**Fig. 2:**
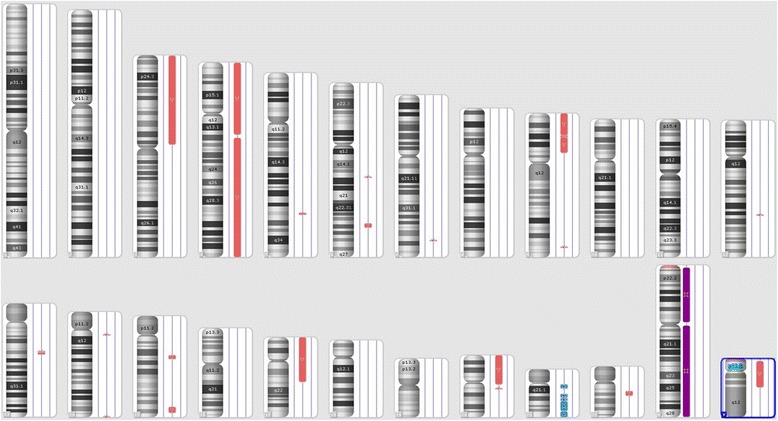
Chromosome view of SNP microarray analysis showing multiple copy number losses

**Fig. 3:**
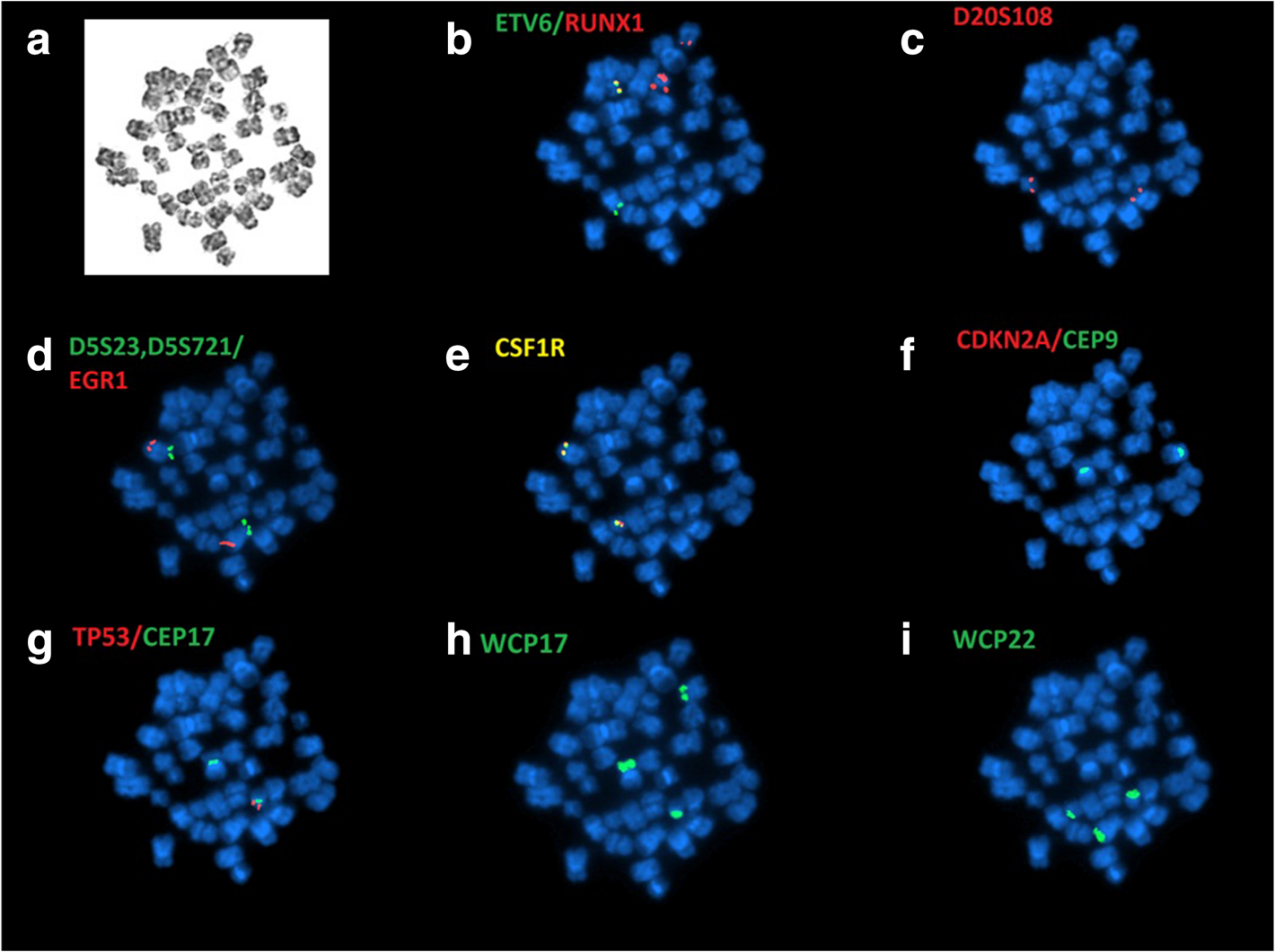
Sequential G-banding and metaphase FISH was performed to refine initial karyotyping result. **a** G-banded metaphase. **b** Metaphase FISH indicated *ETV6* (green) and *RUNX1* (red) fusion as well as *RUNX1* amplification. **c** No deletions for D20S108/20q12 probe in red, one signal on a normal chromosome 20 and the other signal on the derivative chromosome 3. **d** No deletions for D5S23/D5S721(5p15.2) in green and *EGR1* (5q31) in red). **e** No rearrangement for *CSF1R/* 5q33–34, however, one copy was translocated to chromosome 22. **f** Homozygous deletion of *CDKN2A* (9p21) in red; centromere 9 in green. **g** Hemizygous deletion of *TP53* (17p13.1) in red; centromeric 17 in green. **h** Whole chromosome painting (WCP) for 17 (green) stained three different chromosomes, indicated translocations. **i** WCP for 22 (green) stained three different chromosomes, indicated translocations

**Fig. 4:**
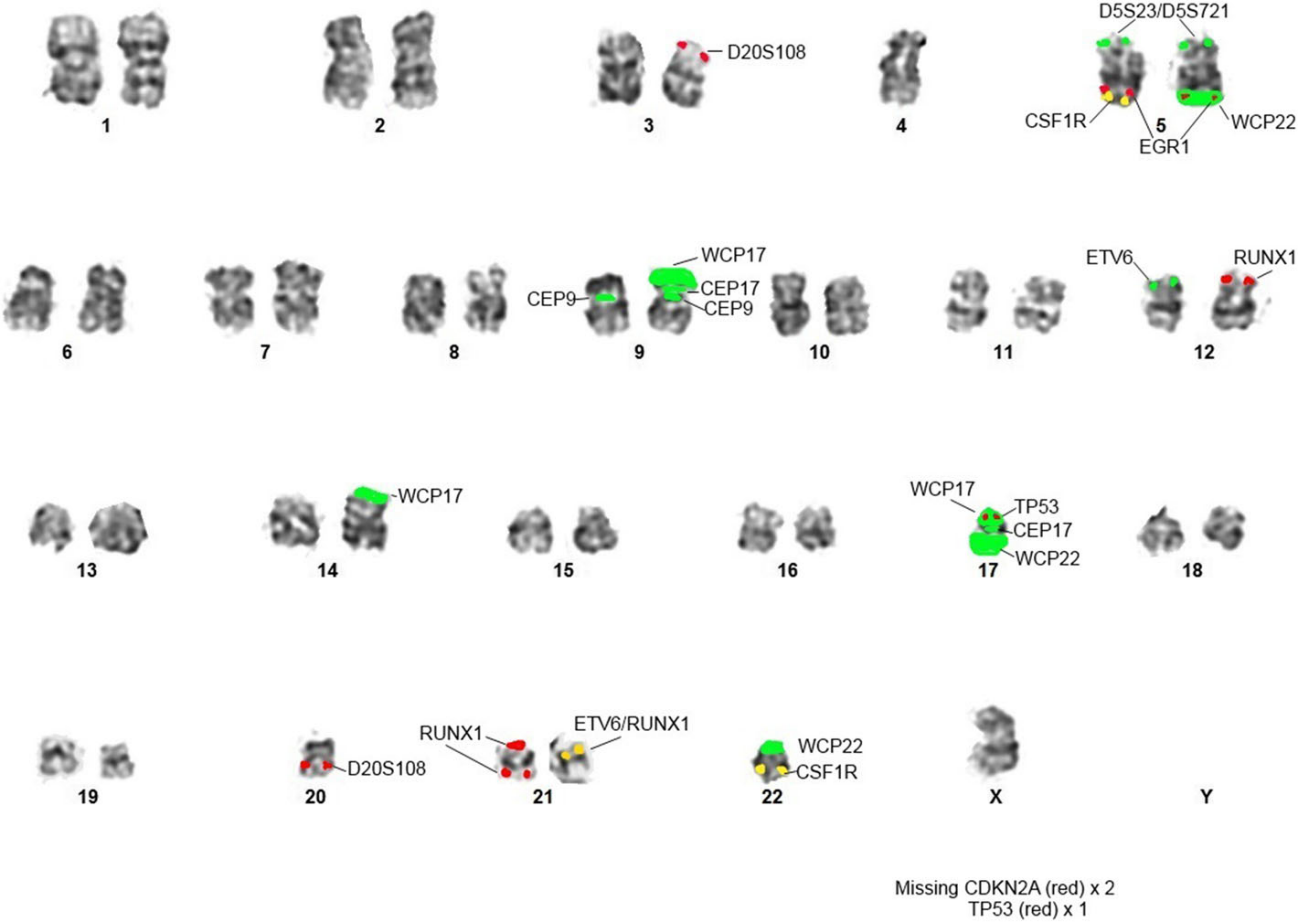
Refined karyotype of abnormal metaphase displayed in Fig. 3a with co-localized FISH signals indicated a hypodiploid clone with 1) a der(3)t(3;20)(p11.2;q11.2)(D20S108+); 2) a der(5)t(5;22)(q32;q11.2)(WCP22+); 3) a del(9)(p13)(CDKN2A-), a dic(9;17)(p13;p11.2)(D9Z1+,CDKN2A-;D17Z1+,TP53-,WCP17+); 4) a t(12;21)(p13;q22)(RUNX1+; ETV6+,RUNX1+) and add(21)(RUNX1+++++); 5) a der(14)t(14;17)(p11.2;q11.2)(WCP17+); 6) a der(17)t(17;22) (TP53+,D17Z1+,WCP17+,WCP22+); 7) der(22)t(5;22)(CSF1R+,WCP22+)

At time of relapse, the complete blood count showed: WBC 2.8 × 10^9^/L, platelets 79 ×10^9^/L and hemoglobin 12.9 g/dL. Bone marrow examination showed 54% blasts. Conventional cytogenetic analysis on the relapsed bone marrow showed a complex karyotype of 41,X,-Y,-3,-4,del(5)(p14),der(5)t(5;22) (q22;q11.2),del(10)(q24q25),-12,-14,-17,add(17)(p11.2),-20,+add(21)(p11.2),der(21)add(21)(p11.2)hsr(?21),der(21)t(12;21)(p13;q22),-22,add(22)(p11.2),+der(?)t(?;5)(?;?)t(?;22)(?;?),+mar[4]/46,XY[15] as initially reported. A nuclear fusion of *ETV6-RUNX1* signal with *RUNX1* amplification were observed in 27.5% of the interphases (Fig. 1). High-resolution SNP microarray revealed losses of chromosomes Yq, 3p, 4, 9p, 17p and 20p, as well as chromothripsis-like pattern of chromosome 21q (Fig. 2). Subsequent metaphase FISH analysis on the G-banded chromosomes targeting *ETV6-RUNX1*, DS523/D5S721/*EGR1, CSF1R, CDKN2A/CEP9, TP53/CEP17* and DS20S108 along with whole chromosome painting (WCP) for chromosomes 17 and 22 (Figs. 3 and 4) showed: 1) a der(3)t(3;20) (p11.2;q11.2)(D20S108+); 2) a der(5)t(5;22)(q32;q11.2)(WCP22+); 3) a del(9)(p13) (CDKN2A-,D9Z1+), a dic(9;17)(p13;p11.2)(CDKN2A-,D9Z1+;D17Z1+,TP53-,WCP17+); 4) a t(12;21)((p13;(q22)RUNX1+; ETV6+,RUNX1+) and add(21)(RUNX1+++++); 5) a der(14)t(14;17)(p11.2;q11.2)(WCP17+); 6) a der(17)t(17;22) (TP53+,D17Z1+,WCP17+,WCP22+); 7) der((22)t(5;22)(CSF1R+,WCP22+) (Table 2). By integrating all the SNP array and chromosome and/or metaphase FISH, the above karyotype was further refined to 41,X,-Y,der(3)t(3;20)(p11.2;q11.2),-4,t(5;22)(q32;q11.2), del(9)(p13),dic(9;17)>(p13;p11.2),t(12;21)(p13;q22),der(14)t(14;17)(p11.2;q11.2), der(17;22)(q11.2;q11.2),-20,add(21)(q22),-22[4]/46,XY[15] (Figs. 3 and 4). In addition, sequencing analysis revealed a 10 base pair deletion-insertion mutation in exon 4 of *TP53* (NM_000546(*TP53*): c.310_321delinsGT p.Q104fs*)*, resulting in a loss of *TP53* function. While this specific mutation is not previously reported in the catalogue of somatic mutations in cancer (COSMIC), this region in exon 4 is known to be involved by similar deleterious (frameshift and truncating) mutations. The patient was treated with blinatumomab and the hyper-CVAD (cyclophosphamide, vincristine, doxorubicin, dexamethasone)/inotuzumab regimen, but only a partial remission was achieved. Due to persistent disease, the patient eventually received a matched unrelated donor allogeneic stem cell transplant (ASCT) 19 months after the initial diagnosis and 6 months after relapse. Unfortunately, the post-transplant course was complicated by liver veno-occlusive disease and relapse of B-ALL. Despite further therapy with R-EPOCH (rituximab, etoposide, vincristine, cyclophosphamide, and doxorubicin) and the patient died 26 months after initial diagnosis.

**Table 2:** G-band, FISH, and SNP-array results comparison

Chromosome	G-band	FISH	SNP-array
Y	-Y	ND^a^	Yp11.31q11.23(2,650,140-28,799,937)x0-1
3	der(3)t(3;20)(p11.2;q11.2)	D20S108+	3p26.3p11.2(61,891-87,302,938)x1-2
4	−4	ND	4q13.3q35.2(73,857,745-190,957,473)x1 ~ 2
5	t(5;22)(q22;q11.2)	wcp22+;wcp22+,CSF1R+	
9	del(9)(p13); dic(9;17)(p13;p11.2)	CDKN2A-,D9Z1+ D9Z1+,CDKN2A-;D17Z1+,TP53-,WCP17+	9p24.3p21.3(203,861-21,608,158)x1 ~ 2, 9p21.3(21,617,251-23,426,271)x1 ~ 2, 9p21.3p13.1(23,436,107-38,787,480)x1 ~ 2,
12	t(12;21)	RUNX1+;ETV6+,RUNX1+	
13	NL^b^	ND	13q14.2(47,925,533-48,966,146)x1 ~ 2, 13q14.2(48,984,342-49,177,989)x1 ~ 2, 13q14.2q14.3(49,182,831-51,194,835)x1 ~ 2
14	der(14;17)(p11.2;q11.2)	wcp17+	
17	der(17)t(17;22)	TP53+,D17Z1+,WCP17+,WCP22+	17p13.3q21.32(525-45,059,684)x1 ~ 2
20	−20	disomy for 20q12(D20S108)	20p13q11.21(61,568-29,497,009)x1 ~ 2
21	add(21)(q22)	RUNX1+++++	21q11.2q21.1(15,006,457-19,142,414)x2 ~ 3, 21q21.2q21.3 (24,806,259-27,905,650)x2 ~ 3, 21q21.3(28,498,600-28,835,696)x2 ~ 3, 21q21.3(29,805,880-31,211,807)x2 ~ 3, 21q22.11q22.2(31,795,184-41,909,261)x2 ~ 3, 21q22.2q22.3(42,364,149-48,097,372)x2 ~ 3
22	-22, t(5;22)	t(5;22)(CSF1R+,WCP22+)	

^a^ND not done

^b^NL normal

## Discussion

We report the case of an 18-year-old with B-ALL associated with iAMP21 and *ETV6-RUNX1*. The patient had a very poor outcome despite intensified chemotherapy and allogeneic stem cell transplant. We also searched the literature and identified six additional cases of B-ALL with co-existing iAMP21 and the *ETV6-RUNX1* [7, 11–14] (Table 3). The median age of these seven patients was 10 years old (range, 2–18) and the median WBC count was 9.1 ×10^9^/L (range, 0.7–34.2 ×10^9^/L). Six of seven (85.7%) cases had karyotypic information with 3 showing an apparently normal karyotype at the diagnosis, likely the result of limited blasts dividing in short-term culture. The remaining 3 cases showed iAMP21 which presented as either the “der(21)” or an “add(21)”; 2 of these cases also had highly complex karyotypes including the current patient. Four of 7 cases had detailed *ETV6/RUNX1* FISH data (Table 3). Case 1 showed the *ETV6-RUNX1* fusion amplification as a sole finding. Patients 2 and 3 apparently showed the *ETV6-RUNX1* fusion as the primary clone and iAMP21 as apparent evidence of a clonal evolution. Interestingly, very similar to the findings as observed in our case (case 7 in Table 3), patient 4 had the *ETV6-RUNX1* fusion only with a normal karyotype at the diagnosis, and had the additional iAMP21 in the relapsed B-ALL. These findings further indicate that iAMP21 is likely a secondary event that results in disease progression. OS information is available for 3 of 7 (42.9%) patients; OS was 34, 28 and 24 months in patients 1, 4 and 7, respectively (Table 3). Patient 1 had a better OS, likely attributable to younger age at the diagnosis. The overall poor prognosis observed in these patients suggests that the adverse clinical impact of iAMP21 overrides the presumably better prognosis associated with *ETV6-RUNX1* in B-ALL.

**Table 3:** Clinicopathologic features of iAMP21 and *ETV6-RUNX1* fusion positive B-ALL cases

Cases	Age(Yr)/Gender	WBC Count (10^9^/L)	BM Blast %	Additional Abnormalities	Outcome	Treatment	References
1	2/M	9.1	NA^a^	Karyotype: 46,XY. FISH: *ETV6-RUNX1* (4–5 copies) fusion in 80% cells	EFS^b^: 22 months. OS^c^: 34 months	ALL-IC-BFM	Case #4 Haltrich, 2013 [12]
2	10/M	NA	NA	*RUNX1* amplification with *ETV6-RUNX1* fusion in 5.5% cells and without *ETV6-RUNX1* fusion in 88.5% cells	NA	NA	Case #4 Ma, 2001 [14]
3	2/F	78	98	*ETV6-RUNX1* fusion with *RUNX1* amplification in 56% cells and without *RUNX1* amplification in 23% cells. Karyotype: 46,XX	NA	NA	Case #23 Mikhail, 2002 [13]
4	7/M	34.2	NA	At diagnosis: *ETV6* deletion with *ETV6-RUNX1* fusion. Normal Karyotype. At relapse: *RUNX1* x 4-5, *ETV6* deletion, *ETV6-RUNX1* fusion. Karyotype: 46,XY,der(21)add(21)(q22)[25]	EFS: 28 months	ALL-BFM’95/ALL-REZ-BFM 2002	Case #1 Haltrich, 2013 [12]
5	11/M	0.7	NA	Karyotype: 46,XY,add(21)(q22)	NA	NA	Case #528 Harrison, 2014 [7]
6	13/M	NA	NA	Karyotype: 44,XY,del(1)(p33),-4,i(9)(q10),-17, t(19;?)(q13.3;?),dup(21)(q?),+1 ~ 2mar[cp16]	NA	NA	Case #530 Harrison, 2014 [7]
7	18/M	2.0	54	At diagnosis: *ETV6-RUNX1* fusion. No Karyotype. At relapse: *RUNX1* amplification (5 copies)/*ETV6-RUNX1* in 27% cells. Complex karyotype.	OS: 26 months	Chemo ASCT^d^	Current Study

^a^ *NA* not available

^b^ *EFS* event-free survival

^c^ *OS* overall survival

^d^ *ASCT* allogeneic stem cell transplantation

In the literature, B-ALL associated with iAMP21 is more frequent than cases of B-ALL with concomitant iAMP21 and *ETV6-RUNX1* fusion. Using an arbitrary age range for adolescents, we summarized 22 cases of B-ALL with iAMP21 for comparison. All these 22 patients had a median age of 15 years at time of diagnosis (range, 13–20) (Table 4) [8, 11, 12, 15, 16] and the male-to-female ratio was 1.75. Most patients had a low WBC count with a median of 3.4×^9^/L (range, 1–15.8). Three (13.6%) patients had *RUNX1* amplification with a normal karyotype; five (22.7%) patients showed a deletion of chromosome 7 as an additional abnormality. Clinical follow up data were available in 20 (90.9%) patients showing a median OS of 29.5 months (range, 9–86 months). Comparing B-ALL with iAMP21 versus B-ALL patients with coexistent iAMP21 and *ETV6-RUNX1*, the iAMP21 only patients had a younger age at disease onset; 9 years old for iAMP21 versus 15 years old for coexistent iAMP21 and *ETV6-RUNX1*, *p = 0.00*. Patients with B-ALL and iAMP21 only also had a higher WBC count; 25×10^9^/L for iAMP21 only patients versus 5×10^9^/L for patients with both iAMP21 and *ETV6-RUNX1*, *p = 0.01*. However, OS was insignificant between these two groups. Although, the clinical data are limited, we believe that patients with B-ALL associated with iAMP21 and *ETV6-RUNX1* can be included in the cytogenetic subgroup of “iAMP21”.

**Table 4:** Clinicopathologic features of iAMP21 positive adolescent B-ALL without *ETV6-RUNX1* fusion

Cases	Age(Yr)/Gender	WBC Count (10^9^/L)	Additional Abnormalities	*RUNX1* Copies/Cell	Outcome	References
1	15/M	3.1	−8,der(16)t(1;16),−21	5–6	CR^b^1 × 9 months	Johnson, 2015 [11]
2	17/M	2.4	Normal Karyotype	>5	Relapsed 58 months after diagnosis following SCT, CR2 × 3 y	Johnson, 2015 [11]
3	20/M	2.2	−13,−14,+21	6–7	CR1 × 30 months	Johnson, 2015 [11]
4	19/M	5.3	del(7)(q11.2)	5–9	CR alive for 2 years	Knez, 2015 [15]
5	13/M	3.7	None	5–10	Unknown	Knez, 2015 [15]
6	15/M	2.1	None	4–8	Relapsed after 7 years of CR	Knez, 2015 [15]
7	13/F	3.0	+X	>5	CR and alive for 3 years	Knez, 2015 [15]
8	15/M	15.8	del(7)(q31)	8	Relapse 2.5 y from diagnosis, CR2 at last chemo block	Haltrich, 2013 [12]
9	14/M	2.2	None	5–10	CR 29 months	Reichard, 2011 [8]
10	15/M	2.0	Not Done	6–8	CR 51 months	Reichard, 2011 [8]
11	13/F	2.8	None	5–10	CR 18 months	Soulier, 2003 [16]
12	17/M	1.0	add(1)(q25)	8	CR 19 months	Soulier, 2003 [16]
13	19/F	10.1	del(7)(p14p21)	6–8	CR 21 months	Soulier, 2003 [16]
14	14/M	2.2	inv(7)(p?15q?21)	5–7	CR 23 months	Soulier, 2003 [16]
15	13/F	3.8	del(7)(q22q35),del(11)(p12)	5	CR 61 months	Soulier, 2003 [16]
16	15/F	9.9	None	4	CR 86 months	Soulier, 2003 [16]
17	15/M	4.3	-Y	4–5	CR 32 months	Soulier, 2003 [16]
18	15/F	NA^a^	add(1)(p?),del(6)(q25)	>4	CR 13 months	Soulier, 2003 [16]
19	13/M	7.6	i(9)(q10),−16	4	CR 18 months	Soulier, 2003 [16]
20	13/F	6.6	add(4)(q31),del(7)(q3?2)	5	CR 10 months	Soulier, 2003 [16]
21	14/M	14.5	Normal Karyotype	6–15	CR 48 months	Soulier, 2003 [16]
22	15/F	NA	Normal Karyotype	15–20	Relapsed	Soulier, 2003 [16]

^a^ *NA* not available

^b^ *CR* complete remission

In addition to the co-existing of *ETV6-RUNX1* fusion and iAMP21, our patient also showed *TP53* deletion with a concomitant *TP53* mutation. *TP53* deletion is frequently observed in B-ALL, particularly in those with hypodiploidy or familial Li Fraumeni syndrome or cancer predisposition syndrome [17]. Sequencing methods allow identification and better characterization of *TP53* mutation in 90% hypodiploid childhood ALL that is important for prognostic assessment [18, 19]. The concomitant *TP53* mutation with deletion could result in “two hits” for *TP53* loss of function and could result in poorer prognosis in our patient [20]. In addition, the null-function of *TP53* or other tumor suppressor gene, such as the homozygous *CDKN2A* deletions observed in our patient, can also promote the chromothripsis of 21q at the genomic level [21]. Hetero- or homozygous deletions of *CDKN2A* are recurrent findings in pediatric ALL. However, they are often considered as secondary events in childhood ALL and increase the likelihood of relapse [22, 23]. In our patient, the *CDKN2A* homozygous deletions were likely following the *ETV6-RUNX1* fusions, to drive disease progression together with the iAMP21.

iAMP21 is also a chromothripsis phenomenal, resulted in the remodeling chromosome 21 in a nonrandom fashion, leading to a stable derivative of chromosome 21 with leukemic potential [6]. Recent studies have provided new insight about the mechanistic events and consequences of chromothripsis [4, 24, 25]. These non-random genomic instabilities of chromosome 21q could be an initial leukemic event [26] in B-ALL pathogenesis, although it was a secondary event in our patient. The additional segmental copy number aberrations involving other parts of the genome, often reflected by complex karyotypes, are likely a secondary event in pathogenesis. High-resolution microarray based testing integrated with traditional chromosome/FISH analysis, particularly the metaphase FISH as performed in our patient, would allow the refinement of the heterogeneous karyotypic findings in iAMP21 B-ALL cases. The clinically critical regions of iAMP21 likely within the 21q22.2-22q22.3 region encoding for 19 to 32 Mb [26–31] in size. These genomic complexities likely contribute to the tumor progression and the poor response to therapy in this subset of the B-ALL patients.

## Conclusions

Our results suggest that the coexistence of iAMP21 and *ETV6-RUNX1* fusion B-ALL is associated with relatively older age, male predominance, and a very poor prognosis. The presence of *ETV6-RUNX1* does not appear to modify the poor prognosis imparted by iAMP21 in B-ALL. Older children with an *ETV6-RUNX1* fusion positive B-ALL should be monitored closely for the development of iAMP21, particularly when a relapse of B-ALL is suspected. Patients with B-ALL associated with both iAMP21 and *ETV6-RUNX1* fit best in the poor prognosis cytogenetic subgroup of “iAMP21”. Integrated genomic testing including high-resolution microarray and metaphase FISH is needed to refine the extremely complex genomic rearrangements.

## Methods

### Flow-cytometry immunophenotypic analyses

Eight- color flow cytometric immunophenotypic analysis was performed according to standard procedures. The panel included antibodies directed against: CD3, CD4, CD5, CD7, CD9, CD10, CD13, CD19, CD20, CD22, CD25, CD33, CD34, CD38, CD52, CD79a, CD117, BCL-2, HLA-DR, myeloperoxidase, IgM (cytoplasmic), kappa and lambda light chains (Becton-Dickinson Biosciences, San Jose, CA, USA), TdT (Supertechs Inc, Bethesda, MD, USA).

### Cytogenetic and FISH analysis

Twenty-four and/or forty-eight hour unstimulated bone marrow cultures were setup for conventional cytogenetic analysis. Using a Leica-microscope imaging system (Leica Microsystems Inc., Chicago, IL) 20 metaphases were examined and karyotypes were prepared according to International System for Human Cytogenetic Nomenclature (ISCN 2013).

FISH studies were performed on cultured bone marrow metaphases and interphases using the probe sets targeting *ETV6/RUNX1*, *BCR/ABL1(ES)*, *MLL, CDKN2A/CEP9,* D5S23/D5S721/*EGR1, TP53/CEP17,* D20S108 (Abbott Molecular, Inc. Abbott Park, IL); and *CSF1R* break-apart (5q32), WCP17, WCP22 (Cytocell Ltd, OGT, UK). A G-banded slide was destained in methanol and hybridized with all the FISH probes above subsequently, according to standard lab procedures. FISH images were then captured in Cytovision and 200 cells were scored by two technologists when applicable.

### SNP microarrays

SNP microarray study was performed using the Affymetrix CytoScan HD array (Affymetrix, Inc. Santa Clara, CA) which contains 2.5 million markers, including 750,000 SNPs and 1.7 million non-polymorphic probes, with extensive coverage over 18,500 RefSeq genes, known cancer genes and 12,000 OMIM genes. In brief, 250 ng of genomic DNA for each NK cell line were hybridized to a CytoScan HD array according to the manufacturer’s protocols. Array data for copy number alterations (CNAs) and copy-neutral loss of heterozygosity (cnLOH) were analyzed using Affymetrix Chromosome Analysis Suite v.3.1 (ChAS) software and the Nexus copy number 7.5 (BioDiscovery Inc, El Segundo, CA) with a reference framework of NA33 (hg19). Regions of copy number alterations larger than 50 markers/400 kb for gain or 20 markers/100 kb for loss and copy neutral loss of heterozygosity (LOH) larger than 3 Mb are recorded. All CNAs were compared with known public databases of normal genomic variants (DGV).

### Molecular study

Nanofluidics-based qualitative multi-parametric reverse-transcriptase polymerase chain reaction (PCR) was performed for the detection of *ETV6-RUNX1* fusion transcripts. PCR-based DNA sequencing was performed to assess for mutations in exons 4 to 9 (codons 33 to 331) of *TP53*.

## Abbreviations

ASCT: Allogeneic stem cell transplant
B-ALL: B cell acute lymphoblastic leukemia
CNAs: Copy number alterations
cnLOH: Copy-neutral loss of heterozygosity
CNS: Central nervous system
CR: Complete remission
DGV: Databases of normal genomic variants
EFS: Event-free survival
FISH: Fluorescence in situ hybridization
iAMP21: Intrachromosomal amplification of chromosome 21
OS: Overall survival
PCR: Polymerase chain reaction
SNP array: Single nucleotide polymorphism microarray
WBC: White blood cell counts
WHO: World Health Organization
